# Benzene formation in electronic cigarettes

**DOI:** 10.1371/journal.pone.0173055

**Published:** 2017-03-08

**Authors:** James F. Pankow, Kilsun Kim, Kevin J. McWhirter, Wentai Luo, Jorge O. Escobedo, Robert M. Strongin, Anna K. Duell, David H. Peyton

**Affiliations:** 1 Department of Chemistry, Portland State University, Portland, Oregon, United States of America; 2 Department of Civil and Environmental Engineering, Portland State University, Portland, Oregon, United States of America; Legacy, Schroeder Institute for Tobacco Research and Policy Studies, UNITED STATES

## Abstract

**Background/Objective:**

The heating of the fluids used in electronic cigarettes (“e-cigarettes”) used to create “vaping” aerosols is capable of causing a wide range of degradation reaction products. We investigated formation of benzene (an important human carcinogen) from e-cigarette fluids containing propylene glycol (PG), glycerol (GL), benzoic acid, the flavor chemical benzaldehyde, and nicotine.

**Methods/Main results:**

Three e-cigarette devices were used: the JUUL^TM^ “pod” system (provides no user accessible settings other than flavor cartridge choice), and two refill tank systems that allowed a range of user accessible power settings. Benzene in the e-cigarette aerosols was determined by gas chromatography/mass spectrometry. Benzene formation was ND (not detected) in the JUUL system. In the two tank systems benzene was found to form from propylene glycol (PG) and glycerol (GL), and from the additives benzoic acid and benzaldehyde, especially at high power settings. With 50:50 PG+GL, for tank device 1 at 6W and 13W, the formed benzene concentrations were 1.9 and 750 μg/m^3^. For tank device 2, at 6W and 25W, the formed concentrations were ND and 1.8 μg/m^3^. With benzoic acid and benzaldehyde at ~10 mg/mL, for tank device 1, values at 13W were as high as 5000 μg/m^3^. For tank device 2 at 25W, all values were ≤~100 μg/m^3^. These values may be compared with what can be expected in a conventional (tobacco) cigarette, namely 200,000 μg/m^3^. Thus, the risks from benzene will be lower from e-cigarettes than from conventional cigarettes. However, ambient benzene air concentrations in the U.S. have typically been 1 μg/m^3^, so that benzene has been named the largest single known cancer-risk air toxic in the U.S. For non-smokers, chronically repeated exposure to benzene from e-cigarettes at levels such as 100 or higher μg/m^3^ will not be of negligible risk.

## Introduction

Electronic cigarettes (“e-cigarettes”) use an electrical resistance coil to vaporize mixtures of propylene glycol (PG), glycerol (GL), nicotine, and flavor chemicals. Vaporization of an e-liquid containing mostly PG and/or GL requires a temperature of ~190 to 290°C: when the ambient pressure is 1 atm, PG boils at ~190°C, GL boils at ~290°C, and PG+GL mixtures will boil between ~190 and ~290°C; the presence of other constituents besides PG and GL (such as water and flavor chemicals) will affect the boiling point. (The presence of significant percentages of other constituents (e.g., water and flavor chemicals) will affect the boiling point.) Temperatures higher than the boiling point of an e-liquid are possible in the coil zone if the rate of e-liquid delivery to the coil does not keep pace with the heat delivery rate: the vicinity of the coil becomes “dry”, and the heat delivery rate surpasses the rate at which heat is carried away by evaporated liquid as “latent heat”.

In general, e-cigarette aerosols tend to be simpler in composition than cigarette aerosols: “e-liquids” are a simpler starting matrix as compared to cigarette filler, and burning cigarettes have been reported to reach 900°C,[1]. Neverthless, multiple toxicants can form upon heating PG and GL.[2–6] Thermal dehydration of PG with loss of one water molecule gives acetaldehyde, and thermal dehydration of GL with loss of two water molecules gives acrolein [2, 6]. Significant amounts of formaldehyde are also possible.[3,4] Kim and Kim [7], using a PG+GL refill fluid (zero nicotine), an unnamed refillable tank device operated, and unspecified settings, reported finding benzene (a known human carcinogen [8,9]) in e-cigarette aerosols at 87.5 μg/m^3^. McAuley *et al*.[10], however, using a simple draw-activated device, reported that benzene was mostly “not found”.

**Fig 1:**
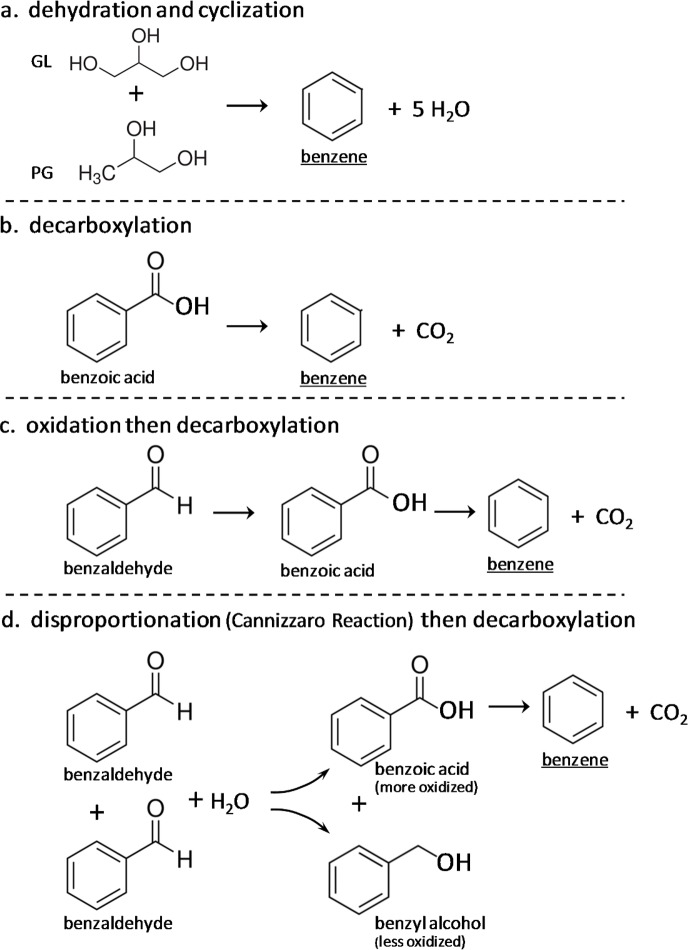
Formation of benzene by four mechanisms: **a.** dehydration according to GL + PG– 5 H_2_O, with cyclization (note: individually, propylene glycol alone and glycerol follow different stoichiometries); **b.** decarboxylation of benzoic acid; **c.** oxidation of benzaldehyde to benzoic acid, followed by decarboxylation (dashed arrow—-> indicates that the exact reaction stoichiometry is not provided); and **d.** disproportionation (Cannizzaro reaction) of benzaldehyde to form benzoic acid + benzyl alcohol.

Dehydration of GL to benzene has been observed [11], and in e-cigarettes a simple dehydration stoichiometry could be PG + GL = benzene + 5 H_2_O (Fig 1A). A second route to benzene in e-cigarettes is decarboxylation of benzoic acid (Fig 1B), and benzene has been known to form when benzoic acid is used as a preservative in beverages.[12] (Benzoic acid has been found by our laboratory in 14 out of 150 e-liquid refill products at levels estimated to be in the range 0.02 to 2 mg/mL, and benzoic acid is an acknowledged ingredient in e-liquids in the JUUL product line.[13]) For a third route to benzene, many aromatic aldehydes are major e-liquid flavor additives, including benzaldehyde (for “cherry”), vanillin, and ethyl vanillin: aldehyde levels as high as several percent (by mass) have been found.[14] Every aldehyde can be oxidized to its corresponding carboxylic acid, which may then undergo decarboxlation. Thus, oxidation of benzaldehyde can give benzoic acid, and therefore, benzene (Fig 1C). For a fourth route to benzene, in what amounts to abiotic fermentation, an aldehyde can undergo redox disproportionation to form a mix of the corresponding alcohol and the acid, and the latter may then undergo decarboxylation. (The acid is more oxidized then the aldehyde, and the alcohol is less oxidized than the aldehyde.) With benzaldehyde, a mix of benzoic acid and benzyl alcohol can then be formed (Fig 1D). (The disproportionation of an aldehyde lacking an “alpha-position” hydrogen atom is the Cannizzaro reaction, which is base-catalyzed (possibly then, by nicotine).)

Herein we describe measurements of gas-phase benzene in e-cigarette aerosols from three types of e-cigarette: a non-refillable e-cigarette (JUUL^TM^), and two variable-power, tank-type devices. For experiments with the tank devices, the fluids used were prepared in the laboratory from PG, GL, benzoic acid, benzaldehyde, and/or nicotine (see Table 1 for compositions). The power settings used for the tank units ranged from “recommended” to beyond. The higher settings were used because they: 1) were accessible by normal use of the devices; 2) may not be “distasteful” to absolutely every user in every use circumstance; 3) will certainly be encountered by users experimenting with settings (as innumerable postings on social media attest); and 4) provide useful information regarding the potential for toxicant formation in e-cigarettes.

**Table 1:** Benzene and Total Particulate Matter (TPM) in E-Cigarette Aerosols Generated Using Two Devices and Different Lab-Prepared Fluids Based on Propylene Glycol (PG) and/or Glycerol (GL). When Together, PG and GL Combined in Equi-volume Amounts. ^13^C-Labelled Compounds Only Present As Indicated.

	conditions				results				
	setting	benzoic acid (mg/mL)	Benzaldehyde (mg/mL)	Nicotine (mg/mL)	number of replicates *N*	benzene produced, μg per g of e-liquid vaped (± 1 SD)	benzene, gaseous, ng/L = μg/m^3^ (± 1 SD)	mg of e-liquid vaped per puff (± 1 SD)	Aerosol log TPM, μg/m^3^ (± 1 SD)
EVOD (Kangertech 1.8 ohm 'Protank'—single horizontal coil with silica wick)									
	PG								
	13W				4	0.40 ± 0.14	59 ± 20	7.4 ± 0.1	8.17 ± 0.00
	GL								
	13W				4	6.6 ± 5.4	1600 ± 1300	12 ± 0.5	8.37 ± 0.02
	PG + GL								
	6W				3	ND	ND	6.8 ± 0.6	8.13 ± 0.04
	13W				3	3.2 ± 1.7	750 ± 390	12 ± 0.2	8.37 ± 0.01
	^13^C PG + ^13^C GL								
	13W				4	1.9 ± 1.5^a^	410 ± 300^a^	11 ± 0.8	8.34 ± 0.03
	PG + GL + benzoic acid								
	6W	9			3	ND	ND	6.7 ± 0.6	8.15 ± 0.04
	13W	9			3	24 ± 12	5400 ± 2600	11 ± 0.1	8.35 ± 0.00
	PG + GL + benzoic acid + nicotine								
	6W	9		12	3	0.08 ± 0.11	9.7 ± 14	6.3 ± 0.3	8.09 ± 0.02
	13W	9		12	3	24 ±14	5200 ± 3000	11 ± 0.2	8.35 ± 0.01
	PG + GL + benzaldehyde								
	6W		10		3	0.16 ± 0.02	21 ± 2.2	6.6 ± 0.9	8.14 ± 0.06
	13W		10		3	23 ±13	5000 ± 2900	10 ± 0.3	8.34 ± 0.01
	PG + GL + benzaldehyde and nicotine								
	6W		10	12	3	ND	ND	6.8 ± 0.5	8.13 ± 0.03
	13W		10	12	3	15 ± 3.3	3300 ± 680	11 ± 0.4	8.35 ± 0.01
Subtank Nano (Kangertech 1.2 ohm ‘OCC’—single vertical coil with cotton wick)									
	PG + GL								
	6W				3	ND	ND	0.5 ± 0.5	6.88 ± 0.37
	13W				3	ND	ND	8.1 ± 2.1	8.19 ± 0.11
	20W				3	ND	ND	17 ± 1.6	8.54 ± 0.04
	25W				3	ND	ND	24 ± 1.4	8.68 ± 0.02
	PG + GL + benzoic acid								
	6W	9			3	ND	ND	1.2 ± 0.2	7.36 ± 0.07
	13W	9			3	ND	ND	9.8 ± 0.3	8.28 ± 0.02
	20W	9			3	ND	ND	20 ± 4.6	8.59 ± 0.09
	25W	9			2	ND	ND	23 ± 0.8	8.65 ± 0.02
	PG + GL + benzoic acid and nicotine								
	6W	9		12	3	ND	ND	0.7 ± 0.4	7.13 ± 0.20
	13W	9		12	3	ND	ND	9.9 ± 0.9	8.29 ± 0.04
	20W	9		12	3	ND	ND	19 ± 0.7	8.58 ± 0.02
	25W	9		12	2	0.11 ± 0.02	66 ± 25^b^	31 ± 6.8	8.77 ± 0.10
	PG + GL + benzaldehyde								
	6W		10		3	ND	ND	1.6 ± 0.3	7.51 ± 0.07
	13W		10		3	0.16 ± 0.01	36 ± 0.6^b^	11 ± 0.4	8.36 ± 0.02
	20W		10		3	0.19 ± 0.03	75 ± 13	18 ± 0.7	8.58 ± 0.02
	25W		10		2	0.16 ± 0.01	101 ± 26^b^	30 ± 8.9	8.79 ± 0.13
	PG + GL + benzaldehyde and nicotine								
	6W		10	12	3	ND	ND	0.8 ± 0.8	7.20 ± 0.36
	13W		10	12	3	0.13 ± 0.01	24 ± 2.1	9.6 ± 0.9	8.28 ± 0.04
	20W		10	12	3	0.14 ± 0.02	52 ± 7.7	19 ± 2.5	8.57 ± 0.06
	25W		10	12	3	0.12 ± 0.01	57 ± 5.2	24 ± 1.2	8.68 ± 0.02

^a^The benzene was ^13^C benzene.

^b^The relative standard for the μg benzene produced per g of e-liquid in the adjacent column differs significantly because of compensating variations in the values of the mass of liquid vaporized for the replicates.

## Materials and methods

### Chemicals and e-cigarette devices

Fully ^13^C-labelled PG and fully ^13^C-labelled GL were obtained from Cambridge Isotopes Laboratory (Tewksbury, MA). Non-labeled PG and GL and standard chemicals were obtained from Sigma-Aldrich Inc. (St. Louis, MO). Determination of benzene levels in the e-cigarette aerosols was by cartridge-based adsorption/thermal desorption (ATD) followed by gas chromatography with detection by mass spectrometry (GC/MS). Three different e-cigarette devices were used in the ATD determinations: 1) JUUL^TM^ personal vaporizer and refill cartridges (“pods”) (Pax Inc., San Francisco, CA) in four different flavors (tobacco, mint, fruit, and crème brûlée) were purchased online in May 2016 (the fluids were analyzed in this study to determine the levels of benzoic acid and nicotine); 2) EVOD^TM^ tank-type atomizer (Kangertech, Shenzhen, China) with 1.8 ohm resistance single horizontal coil and silica wicking material, purchased online in July, 2016; and 3) Subtank Nano^TM^ V.1 (Kangertech) tank-type atomizer with 1.2 ohm resistance single vertical “OCC” single coil and cotton wicking material, purchased online in July, 2016. The JUUL^TM^ system has no user options other than flavor of the cartridge selected. For the EVOD^TM^ and Subtank Nano^TM^ devices: 1) the “recommended” settings were 6W and 10 to 26W, respectively; 2) each replicate aerosol sample for gaseous benzene determination proceeded using a clean tank and a new coil so that any run-to-run changes in benzene production would not be caused by “aging” of the coil etc.; 3) at every wattage setting tested, sample collection was begun 2 h after “conditioning” the new coil; 4) conditioning occurred by taking six 50 mL puffs at 6 W for the EVOD device and 13W for Subtank Nano^TM^ device respectively.

### Sampling

For all three devices, each ATD sample was taken as three or six 50 mL puffs without power (blanks) or with power (aerosol samples) with a puff duration of 5 s and puff-to-puff interval of 60 s. (Regarding the selected puff duration, Hua *et al*. [15] used a data mining exercise for 64 different ENDS users on YouTube to obtain an average puff duration of 4.3 seconds ± 1.4 seconds (SD) for men, and an average of 4.0 seconds ± 0.8 seconds (SD) for women.)

Each ATD sample comprised an average benzene level for six puffs. For the JUUL^TM^ device, blank samples were collected by drawing lab air through an electrically disconnected cartridge. For e-cigarette aerosol sampling, the JUUL^TM^ battery was then connected and device activation occurred automatically as a disposable 60 mL syringe was used to manually pull a puff through a 0.45 μm pore size 28 mm diameter (Phenomenex Inc., Torrance, CA) glass fiber/cellulose acetate (GF/CA) filter (to remove the aerosol droplets), followed by a single ATD gas sampling cartridge containing 100 mg of 35/60 mesh Tenax TA and 200 mg of 60/80 mesh Carbograph 1 TD (Camsco Inc., Houston, TX). All four different JUUL^TM^ flavors were tested. The direct “butt” connections to the filter were held in place using short pieces of flexible 0.125 in. i.d. polyvinyl chloride (PVC) tubing. The benzene levels obtained are minimum values because of the possibility of some small sorptive loss to the PVC pieces; the PVC pieces were replaced after each sample. For the method used (indoor sampling), blank levels corresponded to ~5 μg/m^3^. Final values given are blank-corrected; values not significantly above the blank level are reported as “not detected” (ND).

For the two tank units, the device used for drawing e-cigarette puffs involved a programmable syringe pump. As above, direct “butt” connections were made using short pieces of PVC tubing. Blanks were obtained without activating the power. For vaping, flow for each sample was sequentially drawn through: 1) a GF/CA filter as above; 2) single ATD gas sampling cartridge as above; a 3) three way “T” valve connected to a 1 L Tedlar bag (Model 24633 Supelco Inc. (Bellefonte, PA); and 4) a syringe pump (Model NE-1010, New Era Pump Systems Inc., Farmingdale, NY). Minimal “butt” connections were made as above. For verification of the total sample volume, the puffs were exhausted from the syringe pump back through the three way valve and into the Tedlar bag. Again, final values are blank-corrected, and values not significantly above the blank level are reported as ND.

For confirmation of the formation of benzene in the e-cigarette aerosols by nuclear magnetic resonance (NMR) spectroscopy, aerosol samples were created using an EVOD^TM^ device (as above) with 50:50 PG+GL containing benzoic acid at 1% by weight. Aerosol was generated at 14 W (2 ohms resistance) using five 50 mL puffs, each drawn over five seconds with a puff interval of 1 min. In an approach similar to that described by Jensen et al. [4], the aerosol was drawn into a 1 mL septum vial containing 600 μL of DMSO-d6. The flow inlet to the vial was an 18 gauge needle, as was the flow outlet. (Gas-phase benzene will be captured efficiently by this method.) Samples were run using a 600 MHz NMR spectrometer.

### Mass of e-liquid vaped

Values for mg of e-liquid vaped per puff (and for the resulting TPM concentration) were estimated based on weight loss of the e-cigarette unit for each run.

### Gas-phase benzene

For all the e-cigarette aerosol samples, >89% of the benzene can be deduced to have been in the gas phase as follows: 1) As discussed by Pankow *et al*.[16,17] the percent of a compound in the gas phase of an aerosol is given by *P*(%) = 100%/(1+*K*_p_TPM) where *K*_p_ (m^3^/μg) is the gas-to-particle (*i*.*e*. droplet) partition coefficient, and TPM is the suspended “total particulate matter” level for the aerosol. 2) It can be estimated that *K*_p_ ≈ 10^−9.7^ m^3^/μg for benzene at 20°C for partitioning to e-cigarette aerosol particles (as based on the approach of Pankow *et al*.,[16,17] using a benzene vapor pressure of 0.099 atm at *t* = 20°C,[18] an activity coefficient for benzene (ζ_benzene_) in glycol solutions of ~14 [19], and an average molecular weight of $\bar{\text{MW}}$ ≈ 84 g/mol for a 50:50 by volume PG:GL mixture). 3) For all the aerosols created here, TPM ≤ 10^8.8^ μg/m^3^ (= aerosolized liquid mass/total puff volume). Then *P*(%) ≥ 100%/(1+10^−9.7^10^8.8^) = 89%.

### ATD cartridge analyses by GC/MS

For each standard sample for method calibration, an ATD cartridge was charged with 2 or 4 μL of a 1 to 100 ng/μL solution of benzene in methanol, followed by a 50 mL/min flow of nitrogen for 10 min. Each ATD sample cartridge and each standard ATD cartridge was thermally desorbed using a TurboMatrix 650 ATD unit (PerkinElmer, Waltham, MA). Prior to desorption, the ATD unit automatically amended each cartridge with 20 ng of fluorobenzene as the internal standard compound. Each ATD cartridge was thermally desorbed for 10 min at 285°C with helium desorption flow of 40 mL/min and split flow of 20 mL/min. The desorption stream was trapped at -10°C on the intermediate “air monitoring trap” (ATP). The ATP was then thermally desorbed at 295°C and 25 psi constant pressure helium with a split flow of 8 mL/min for 4 min. The non-split portion of the desorption gas stream passed onto the GC column which was mounted in an Agilent (Santa Clara, CA) 7890A GC. The GC was interfaced to an Agilent 5975C MS operated in electron impact ionization mode. The MS scan range was 34 to 400 amu. The electron multiplier voltage was 1400 V. The fused silica capillary GC column was a model Rxi-624Sil MS (Restek Inc., Bellefonte, PA) of 30 m length, 0.25 mm i.d., and 1.4 μm film thickness.

### JUUL^TM^ pod analyses by LC/UV

JUUL^TM^ pods were opened to obtain the e-liquid for determination of the benzoic acid and nicotine concentrations. Aliquots of 100 μL were withdrawn, diluted 1:100 with methanol, and filtered with a syringe-mounted PVDF (polyvinylidene fluoride) filter, 13 mm diameter, 0.22 μm pore size, obtained from Thermo Fisher Scientific, Inc. (Waltham, MA). Analyses proceeded by high performance liquid chromatography (HPLC) using an injection volume of 20 μL, a Waters Corp. (Milford, MA) Model 1525 binary solvent delivery module (with Rheodyne 7725i injector), a Discovery™ C-18 column (250 × 4.6 mm × 5 μm, Supelco Inc.) at 40°C, and a Waters Corp. Model 2996 photodiode array detector. The mobile phase composition was maintained isocratic, 40:60 methanol:water amended with 0.1% trifluoroacetic acid (1 mL/min). The detector wavelength for benzoic acid was 228 nm, and 259 nm for nicotine. The calibration ranges for the injected standards were 0 to 1 mg/mL for benzoic acid, and 0 to 0.1 mg/mL for nicotine.

## Results and discussion

### Benzoic acid and nicotine levels in JUUL^TM^ pod fluids

The concentrations of benzoic acid and nicotine in the JUUL^TM^ pod fluids were found to be 44.8 ± 0.6 and 61.6 ± 1.5 mg/mL respectively, corresponding to a benzoic acid/nicotine molar concentration ratio of 0.97 to 1). For comparison, as noted above, analyses in our laboratory have indicated the presence of benzoic acid in 14 commercial refill e-liquids at levels estimated to be in the range 0.02 to 2 mg/mL.

### Benzene confirmation by NMR

Benzene formation (^12^C_6_) was confirmed by NMR for e-cigarette aerosol collected in DMSO-d6 and generated with the EVOD^TM^ device operated at 14 W (2 ohms resistance) using 50:50 PG+GL (both ^12^C_3_) containing benzoic acid (^12^C_6_) at 1% by weight. The expected strong singlet peak in DMSO-d6 at 7.37 ppm [20] was observed; addition of authentic benzene caused the peak to increase without introduction of other peaks.

### Formation of ^13^C_6_ benzene From 50:50 PG+GL (both ^13^C_3_)

Formation of benzene with all carbons ^13^C (i.e., ^13^C_6_ benzene, MW = 84) was confirmed to occur from 50:50 PG+GL (both ^13^C_3_) (see Table 1) at levels consistent with the analogous runs with 50:50 PG+GL (both ^12^C_3_) (see Table 1).

### Gas-phase benzene levels measured in e-cigarette aerosols

**Fig 2:**
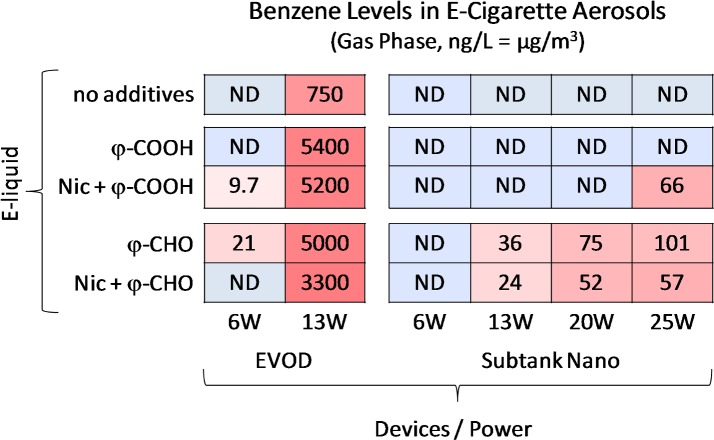
Benzene levels in e-cigarette aerosols generated with two different devices, different power levels, and 50:50 propylene glycol:glycerol with and without nicotine, benzoic acid, and/or benzaldehyde.

Gas-phase benzene was not found above blank levels in any of the JUUL^TM^ samples for any of the four flavors. For the other two devices, benzene gas-phase concentrations and deliveries (as μg benzene per g e-liquid vaped) are summarized in Fig 2, with other details provided in Table 1. Benzene formation was found to be very strongly dependent on device. With 50:50 PG+GL, for the EVOD device at 6W and 13W, the mean concentration values were 1.7 and 750 μg/m^3^, respectively. For the Subtank Nano device, at 6W and 25W, the mean values were remarkably lower, namely ND and only 1.5 μg/m^3^, respectively.

Significant formation from benzoic acid and benzaldehyde was observed for both the EVOD and Subtank Nano devices at the high power settings used. With benzoic acid and benzaldehyde at 9 to 10 mg/mL, for the EVOD device, values at higher power levels were as high as 5000 μg/m^3^. Remarkably, the values at the higher power levels for the Subtank Nano device were much lower, ≤ ~100 μg/m^3^.

### Comparisons with benzene levels in ambient air and cigarette tobacco smoke

For tobacco smoke from “regular” cigarettes, the 1999 Massachusetts Benchmark Study [21] reported an average benzene delivery of 86 μg/cigarette. Assuming a total puff volume of ~400 mL/cigarette for the smoking protocol used [22], such deliveries correspond to a smoke benzene concentration of ~200,000 μg/m^3^, and so a much higher risk *from benzene* for chronic use of tobacco cigarettes as compared to e-cigarettes. However, median ambient air concentrations of benzene in locations in the U.S. in 2013 were ~1 μg/m^3^ [23]. Levels such as these resulted in benzene being named by the 2002 National-Scale Air Toxics Assessment (NATA) as the largest single known cancer-risk air toxic in the U.S.[24]. It can therefore be concluded for non-smokers that chronically repeated exposure to benzene from e-cigarettes at levels such as 100 μg/m^3^ will not be of negligible risk.

## Supporting information

S1 Text Information on e-cigarettes used. (DOCX)
