# Supplementary material for: Benzene formation in electronic cigarettes
Source: PLoS One. 2017 Mar 8;12(3):e0173055. doi: 10.1371/journal.pone.0173055 (PMC5342216; doi:10.1371/journal.pone.0173055)
Supplement: S1 Text — (DOCX) [file pone.0173055.s001.docx]

**Figure S1: Atomizers Used**


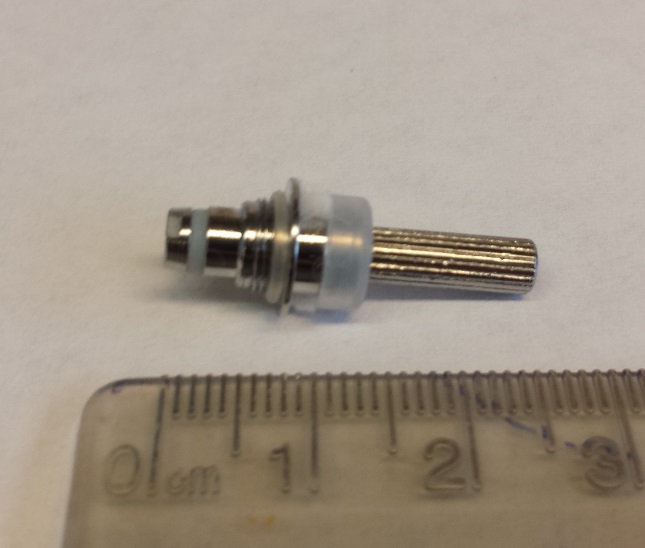

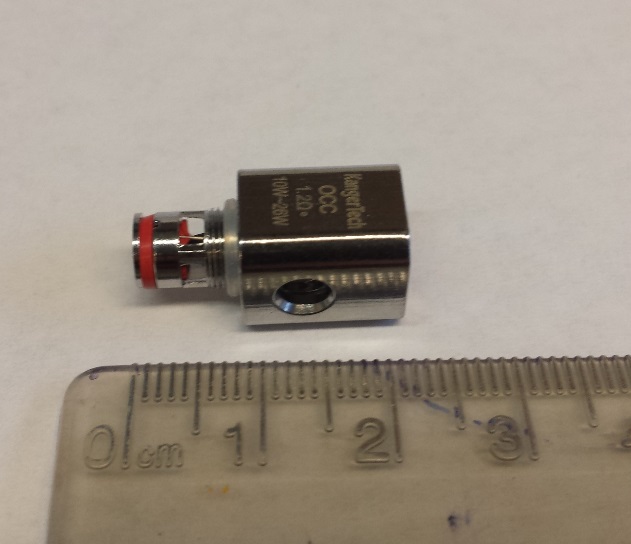


EVOD (left) and SubTank (right) replacement atomizers

EVOD - Kangertech 1.8 ohm 'Protank' - single, horizontal coil. Silica wicking material.

Subtank Nano - Kangertech 1.2 ohm 'OCC' - single, vertical coil. Cotton wicking material

|  | **EVOD** | **SUBTANK** |
| --- | --- | --- |
| **Bottom (air flow in)** |  |  |
| *Area (mm^2^)* | 2.14 | 23.22 |
| **Top (air flow out)** |  |  |
| *Area (mm^2^)* | 7.91 | 31.66 |
| **Mass of Coil Housing** |  |  |
| *Mass (g)* | 2.08 | 9.45 |

Compared to the Subtank system, the EVOD atomizer has ~5 times less coil housing mass (~2.1 g EVOD, 9.5 g Subtank) and ~10 times less 'airflow cross-sectional area' (~2 mm^2^ EVOD, ~23 mm^2^ Subtank).

The orientation of the heating coil and wicking material affects the wick re-wetting rate (how quickly the wicking material becomes saturated with eliquid after puffing.) The EVOD coils used have an air space around the horizontally oriented coil where the aerosol mixture is evolved. Conversely, vertical OCC coils draw air through the heating element with wicking material on the outside. E-liquid has a shorter distance to travel from reservoir to heating element in vertical OCC coils than in horizontal EVOD coils.
